# Mechatronic Wearable Exoskeletons for Bionic Bipedal Standing and Walking: A New Synthetic Approach

**DOI:** 10.3389/fnins.2016.00343

**Published:** 2016-09-29

**Authors:** Gelu Onose, Vladimir Cârdei, Ştefan T. Crăciunoiu, Valeriu Avramescu, Ioan Opriş, Mikhail A. Lebedev, Marian Vladimir Constantinescu

**Affiliations:** ^1^Department of Physical and Rehabilitation Medicine, University of Medicine and Pharmacy “Carol Davila”Bucharest, Romania; ^2^Teaching Emergency Hospital “Bagdasar-Arseni”Bucharest, Romania; ^3^Research and Technological Design Institute for Machines ConstructionBucharest, Romania; ^4^Miller School of Medicine, University of MiamiMiami, FL, USA; ^5^Department of Neurobiology, Duke UniversityDurham, NC, USA; ^6^Holistic Dental Medicine Institute – ROPOSTUROBucharest, Romania

**Keywords:** mechatronic, portable/wearable exoskeletons, bipedal standing, bionic walk assistance, models

## Abstract

During the last few years, interest has been growing to mechatronic and robotic technologies utilized in wearable powered exoskeletons that assist standing and walking. The available literature includes single-case reports, clinical studies conducted in small groups of subjects, and several recent systematic reviews. These publications have fulfilled promotional and marketing objectives but have not yet resulted in a fully optimized, practical wearable exoskeleton. Here we evaluate the progress and future directions in this field from a joint perspective of health professionals, manufacturers, and consumers. We describe the taxonomy of existing technologies and highlight the main improvements needed for the development and functional optimization of the practical exoskeletons.

## Human posture and walking, and their restoration to the disabled

Vertical posture and bipedal gait are two hallmarks of human biomechanics and motor physiology, which emerged over the millions of years of evolution. While our body size and overall physical performance are not particularly impressive compared to other animal species, having our hands relieved from body support against gravity gives us a number of advantages, dexterity being one of the most important ones. In addition to motor physiology, virtually any human physiological function is adapted to the bipedal posture and walking, for example breathing, digestion, and excretion (Uebelhart et al., 2000)^1^. Moreover, human bipedalism has had a great influence on our daily social interactions and the manmade habitat.

While the surrounding objects and structure of the buildings all fit the needs of healthy humans, the conditions are quite different for disabled, wheelchair bound people. No matter how many facilities would be made in order to overcome their numberless structural barriers, new hurdles are always to be expected. Wheelchair users will never be able to completely adapt to the habitat built for the people who walk bipedally and stand upright. By contrast, the cartoon of a person in the wheelchair is the worldwide used symbol for handicap.

Although there is no cure for many lesions of the spinal cord and the brain (Talley Watts et al., 2015)—the major causes of paralysis—essential improvements can be made to the quality of life of handicapped people using devices that assist vertical stance and bipedal walking (Louie et al., 2015). These assistive technologies are based on mechatronic, robotic, and bionic exoskeletons (Chen et al., 2013). Such devices clearly represent one of the most important developments in rehabilitation of paralysis, even though they are currently not expected to deliver a truly spectacular, translational progress. Rather, they are considered as viable intermediary solutions, which bring us closer to the long-awaited recovery of posture and gait to the paralyzed patients. While much work is still needed to improve these external aids, they already have shown effective daily use capabilities.

## Assistive technologies: manufacturer and consumer perspectives

In the last decade and mostly in its second half, a series of exoskeletons have been designed, built, clinically tested in small groups of subjects, and reviewed in the literature^2^^−^^9^. These previous publications have fulfilled the promotional and marketing objectives of the manufacturers, and several exoskeletons are currently commercially available. Yet, an optimal, fully functional assistive device has not yet emerged from this research and development^10^^,^^11^ (Chen et al., 2013; Arazpour et al., 2015b; Lajeunesse et al., 2015; Louie et al., 2015).

An optimal, practical exoskeleton should fulfill the following requirements:

- The system should be safe.
- It should be effectively wearable11 in the common sense of the term, mainly regarding don and doff issues. Additionally, it should be psychologically acceptable in terms of self-esteem, miniaturization, and esthetics. Ideally, the exo-suit should be thin and wearable like clothes or underwear.
- The exoskeleton should be appropriate for long time performance, including the performance in community (Louie et al., 2015).
- It should produce very low, practically imperceptible noise when functioning.
- The assistive device should be truly affordable and cost effective.

**Figure 1:**
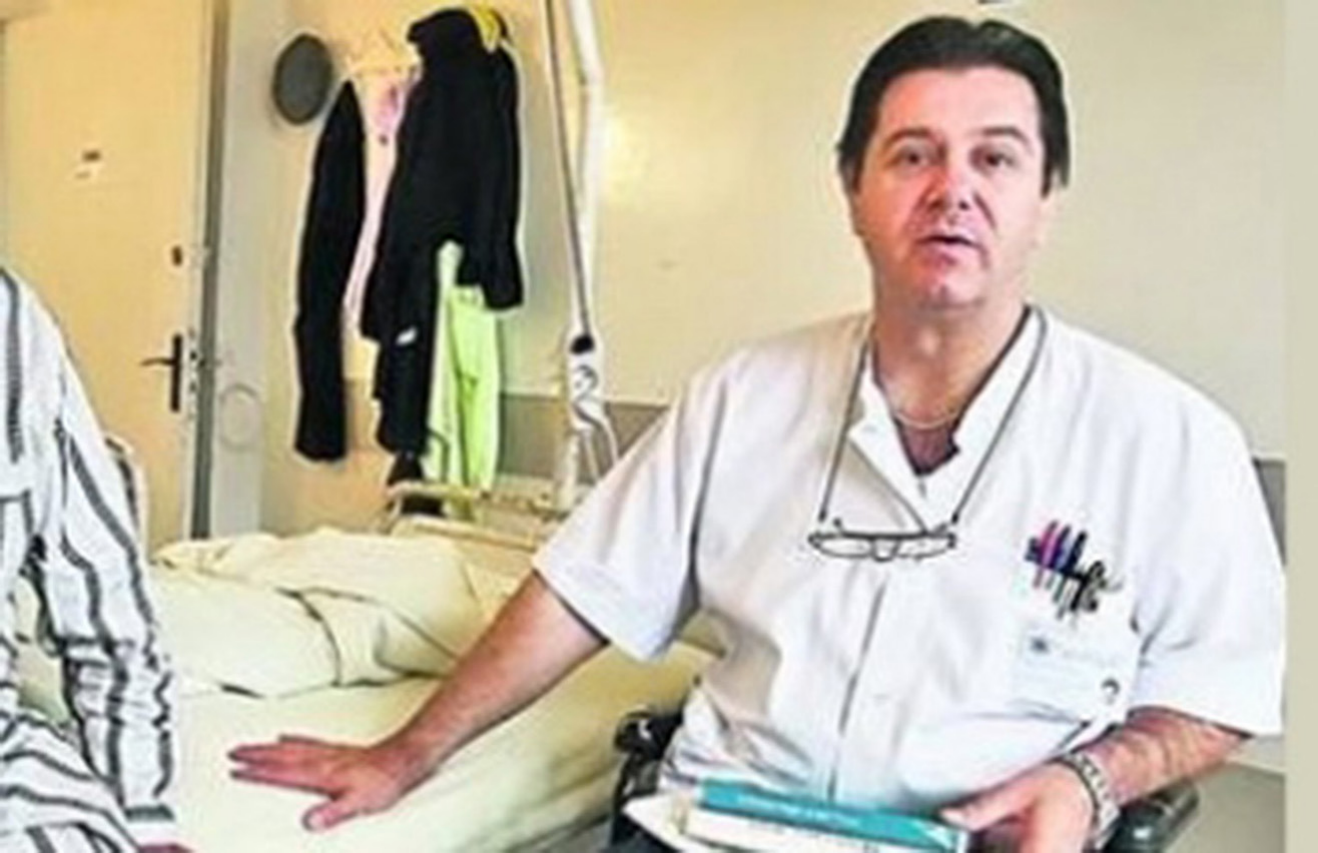
**The picture of the first author Prof. Dr. Gelu Onose in wheelchair visiting patients in the NeuroRehabilitation Clinic Division of the ‘Bagdasar-Arseni’ Teaching Emergency Hospital, Bucharest, Romania**. Reproduced with permission of the TV producer, Romanian Television.

We believe that achieving these requirements should be the main goal of the field, so we focus on these practical, consumer-oriented objectives in this article. We start with proposing a taxonomy of exoskeletons. We then discuss the improvements of these assistive devices that need to be achieved in near-term and long-term. Our views reflect both the professional, multi-disciplinary expertise of the authors and consumer perspective. The first author (Figure 1) sees both sides of the coin: he is an academic physician in Physical and Rehabilitation Medicine (with special focus on Neurorehabilitation) and in Gerontology and Geriatrics, and also a chronic, complete paraplegic, dependent on the wheelchair.

Our multidisciplinary approach intends—without underestimating the legitimate competition between different technical solutions and producers—to foster the optimization of assistive and rehabilitation devices, so that they could eventually become suitable and available to as many people in need as possible. Being one of the consumers too, the first author is looking forward to welcome this long awaited accomplishment, no matter which exoskeleton model meets the expectations first.

## Main indications

The currently available wearable powered exoskeletons serve two main functions. The first function is medical: providing assistive and rehabilitation technology for disabled people^12^. The second function is non-medical: augmenting normal human physical capabilities.

As to the non-medical domain, we are covering it here only briefly. Noteworthy, non-medical exoskeletons have indirect linkages to medical issues, such as contributions to technology development and prevention of occupational injuries. The other uses encompass military devices for enhancement of soldier speed, power, and interactions with the equipment^13^^−^^15^; exoskeletons for people professionally involved in critical rescue situations, such transportation of wounded individuals to safety from the war or civil disaster areas^13^^−^^16^; civil applications for lifting and carrying heavy objects and patients^17^; and exoskeletons that augment human gait by assisting leg muscles and supporting a comfortable orthostatic posture. The target population for these applications includes the elderly with weak legs, personnel engaged in long-distance walking and/or performing flexion and extension movements with their lower limbs, and people utilizing exoskeleton appliances for entertainment^18^.

The medical domain includes wearable devices utilized for prophylactic, rehabilitation, and assistance to disabled people. Taxonomy of these devices reflects the needs of different types of patients. A complete paraplegic would need a device that performs a set of sophisticated tasks, and there is currently no device with these capabilities. Such a device would require no effort from the paralyzed patient, and would do almost everything for the patient.

The device for a complete paraplegic would have to ensure:

- Complete assistance to orthostatic posture and walking, including the very difficult task of maintaining balance in order to avoid falls with severe consequences (Kannape and Lenggenhager, 2016).
- Prevention of spasticity and contractures (Arazpour et al., 2013) and related articular stiffness and pain.
- Prevention of venous and lymphatic stasis in the lower limbs, which could result in lower extremity edema and related complications due to prolonged standing (Louie et al., 2015).
- Partial compensation of the body weight to avoid injuries, such as fracture of the talus (Louie et al., 2015).

The complete paraplegia is classified by the scales of American Spinal Injury Association (ASIA) and International Spinal Cord Society (ISCoS) (“Standard Neurological Classification of Spinal Cord Injury”^19^, Frankel et al., 1969). Mechatronic wearable exoskeletons can be very useful as assistive devices in these cases because in complete chronic lesions of the spinal cord, as a rule, there are not enough remaining neural-muscular units to be trained. The possibility that exoskeletons could be used for rehabilitation in such cases, for example for rehabilitation of muscle activity, is still unclear (Arazpour et al., 2015b). The use of exoskeletons still can induce neuroplasticity (Muresanu et al., 2012; Bryce et al., 2015; Louie et al., 2015) that improved patient interaction with the device^20^. Additionally, a supplementary input from the patient's collected and decoded cortical activity might improve the man-machine interaction and the overall functional outcome.

The indications are different for incomplete paraplegics classified as AIS/ Frankel B-D, tetraplegics with low cervical spinal cord lesions classified as AIS/ Frankel C, and for hemiplegics and hemiparetics with fully preserved functionality of the non-affected hemi-body and limbs, and no sensorial, cognitive and balance serious impairments. The other potential beneficiaries of such exoskeletons are patients with neuromuscular and somatic impairments, including patients with polyneuropathia in lower limbs (Zeilig et al., 2012), multiple sclerosis, Parkinson's disease, hip or knee osteoarthritis^21^, limb fractures, and amputated limbs (Chen et al., 2013).

In hemiplegia cases, an H2 (Technaid S.L., Spain) robotic exoskeleton is more appropriate for gait rehabilitation (Bortole et al., 2015)—an exoskeleton based on two control strategies: (i) adaptive trajectory control for guiding the patient's limb within a desired path, the strategy that facilitates patient interaction with the device, and (ii) admittance control strategy that captures the user's movements during assistive training phase and reproduces it during active training phase (Bortole et al., 2013). The main feature of the wearable exoskeletons indicated to approach these kinds of patients is usually not an assistive control of the trunk. An essential feature of using such devices is training induced rehabilitation, where the exercise outcomes are compared to the time spent exercising (Louie et al., 2015).

The choice of the exoskeleton system and training regime critically depend on the extent of neurological impairment, the affected neural site (central, peripheral, or both), and the state of muscles (which can change with training). Additionally, it is important to take into account and correct by training such conditions as pain, contracture, osteoporosis, bed sores, and edema (Arazpour et al., 2013).

In the disabled elderly, the degree and characteristics of multimorbidity should be considered (Salive, 2013), particularly balance disorders, cardio-respiratory impairments, and para-physiological sarcopenia (Morley, 2012). The assistive and rehabilitation devices should be adjusted to these conditions. For instance, if an older adult has preserved, although altered, ability to stand-up and walk, extended trunk stabilization may not be needed. However, if the senior beneficiary has marked sensory and/or balance impairment, a maximally assistive devices might be more appropriate, in order to avoid frequent falls in the older population^22^. The frequency of falls is variable (6–34%) in different countries and regions: (Kalache et al., 2007). Falls represent some of the most serious negative events with considerable pathological consequences, including disabilities and life threatening conditions. Such negative events result in economic burden for the patients, their families, and the society^22^ (Kalache et al., 2007). These considerations will become progressively more important in the future, as the global process of demographic ageing intensifiers^22^ (Kalache et al., 2007; Chen et al., 2013).

## Current technologies

Currently popular wearable powered exoskeletons include Isocentric Reciprocating Gait Orthosis (IRGO), Hybrid Assistive Leg/Limb (HAL), ReWalk, Ekso, Mina, Wearable Power-Assist Locomotor (WPAL), Rex, Indego, Keeogo, Kickstart, Stride Management Assist, and ExoAtlet^23^. These devices have been recently reviewed^11^ (Arazpour et al., 2013, 2015a,b; Chen et al., 2013; Lajeunesse et al., 2015; Louie et al., 2015). These review articles documented the following characteristics of the existing systems:

- Benefits, including compared to the robotic exoskeletons fixed to treadmills (Chen et al., 2013; Louie et al., 2015).
- Safety (Lajeunesse et al., 2015).
- Efficacy in restoring walking^11^ (Louie et al., 2015), including the performense in community settings (Lajeunesse et al., 2015).
- Clinical effectiveness outcomes, imcluding such parameters as stair climbing performance, speed, mobility, quaility of life, and independence^11^ (Chen et al., 2013; Lajeunesse et al., 2015; Louie et al., 2015; Arazpour et al., 2015a,b; Lajeunesse et al., 2015).
- Walking distance (Benson et al., 2016).
- Vertical ground reaction force (vGRF) that quantifies the pattern and magnitude of mechanical loading (Fineberg et al., 2013). This characteristic is particularly important for exoskeletons used by completely paralyzed persons.
- Training protocols (Lajeunesse et al., 2015; Louie et al., 2015).
- User satisfaction and secondary benefits and skills achieved with the exoskeleton (Lajeunesse et al., 2015).
- Level of scientific evidence (Lajeunesse et al., 2015).
- Cost-effectiveness outcomes^11^.

Overall, the current literature reports both successes of exoskeleton technology and remaining problems. One important achievement is that wearable exoskeletons enable patients with complete paralysis of the lower part of the body to stand and walk. The speed of walking is modest; it varies with the level of injury and training duration (Louie et al., 2015). In addition to paraplegics, wearable exoskeletons can be used by patients with post-stroke hemiparesis (Bortole et al., 2015), as well as non-stroke neurological pathologies^24^ (Esquenazi et al., 2012), such as cerebral palsy, myelome-ningocele, traumatic brain injury and Guillain Barré syndrome (Zeilig et al., 2012).

Wearable exoskeletons have advantages compared to Hip-Knee-Ankle-Foot-Orthoses (KHAFO). For example, powered gait orthosis (PGO) and the IRGO outperform HKAFO, as evident from the improvements in physiological cost index (PCI), distance walked, and walking speed (Arazpour et al., 2013). Additionally, wearable exoskeletons have certain advantages compared to the treadmill-based ones. With wearable exoskeletons, patients get much greater autonomy. The autonomy in turn facilitates rehabilitative training because the device can be used at home in addition to specialized facilities (Chen et al., 2013; Louie et al., 2015). Yet, autonomous use of wearable exoskeletons in community settings still has to be demonstrated (Lajeunesse et al., 2015). Overall, there is still a discrepancy between the consumer high expectations of autonomy and versatility and the actual effectiveness of using wearable exoskeletons (Benson et al., 2016).

A critical analysis of the assessments of exoskeletons as health technology^11^ indicates the need for more research, particularly regarding long-term performance (Lajeunesse et al., 2015), assessment of training (Louie et al., 2015), randomized controlled trials, and economic analysis11. Overall, the level of scientific evidence remains low in clinical trials of exoskeletons (Lajeunesse et al., 2015). Prevention and detection of medical complications in the exoskeleton users is yet another area that needs more research (Benson et al., 2016). A unified framework is needed for assessment of exoskeleton performance (Bryce et al., 2015). Such a framework is needed to make different clinical trials comparable. The framework should include at least six modules: functional applications, personal factors, device factors, external factors, activities, and health outcomes (Bryce et al., 2015).

**Figure 2:**
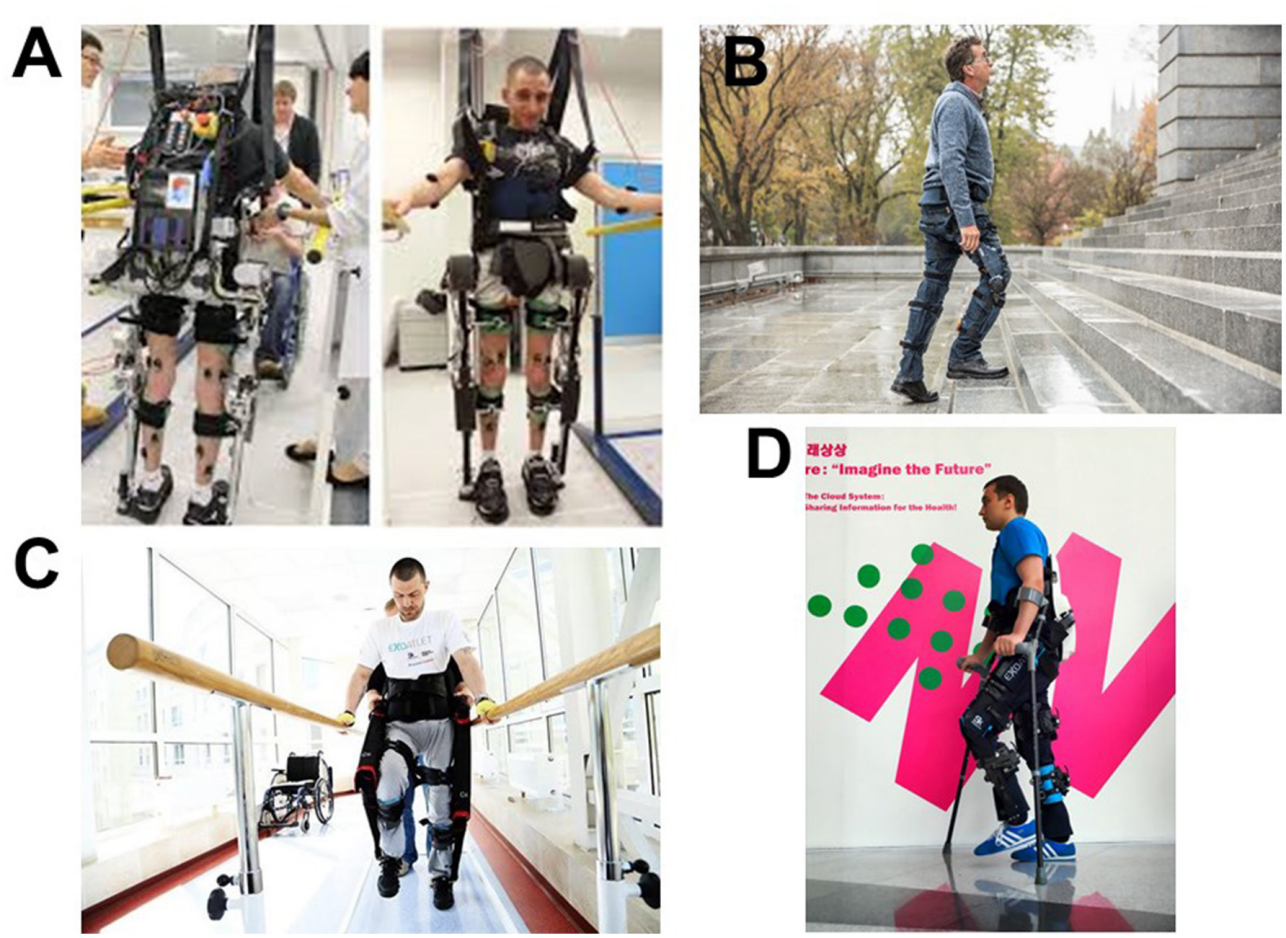
**Images of several types of exoskeletons**. **(A)** Mindwalker mind-controlled exoskeleton could help the disabled walk again. With courtesy from Professor van der Kooij at University of Twente, NL. **(B)** The Keeogo™ exoskeleton from B- TEMIA reveals the latest in an increase of the human systems (human augmentation systems) designed to help people walk more and better. Keeogo™ eliminates several problems in patients with Parkinson's disease. With courtesy from Danielle Beaudoin at b-termia.com. **(C,D)** The ExoAtlet is a powered exoskeleton designed to assist patients during their rehabilitation after stroke, injury, or unsuccessful operation. ExoAtlet automatically repeats the natural patterns of walking, has electrical stimulation system, and physiological sensors. The control system of the ExoAtlet is unique: it collects data from body angles, allows to set the height and length of the step, which provides: (i) standing still; (ii) classic walking; (iii) walking on angled surface; (iv) stepping over obstacles; and (v) comfortable walking up & down stairs. ExoAtlet can be used in rehabilitation centers and at home^24^. ExoAtlet can be controlled with the app on tablet when used in clinics. Experienced user of ExoAtlet use “thinking” crutch for control. With courtesy from Ekaterina Bereziy at exoatlet.ru.

Current exoskeletons (Figure 2) provide several secondary skills and benefits (Lajeunesse et al., 2015). Exoskeleton with compact design and small backpacks are particularly useful in this respect (Chen et al., 2013) because they allow don, doff and wearing an exoskeleton while sitting in a wheelchair. These operations are possible using electrically actuated ReWalk and ExoAtlet, hydraulically actuated Ekso, and Indego, which combines electriaclly actuated hip and knee joints for both legs (Chen et al., 2013) with functional electrical stimulation (FES) of paralyzed, yet responsive muscles^23^ (Chen et al., 2013). This combination of the exoskeleton action with FES training improves rehabilitation results.

## Future directions

Notwithstanding significant technological advancements in the field, the current exoskeleton systems still need much improvement for them to become practically efficient. The areas that need improvement remain almost the same as about one decade ago. Accordingly, our assessment made 8 years ago (Onose et al., 2008) remains of current interest: exoskeletons need further miniaturization, optimization of actuators and sensors. Ideally, exoskeletons should become robotic orthotic suits wearable beneath the clothes.

**Figure 3:**
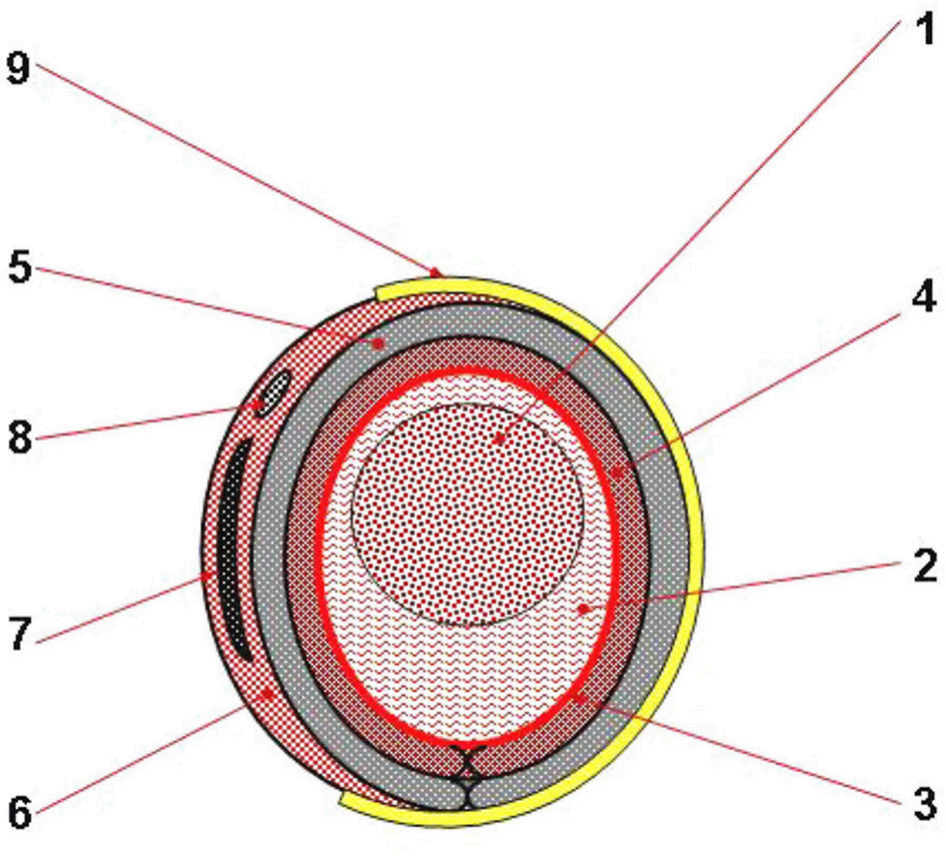
**Concept design of a cross section through lower limb(s)'module of the MOD**. 1, Bony area; 2, Muscle area; 3, Skin and sucutaneous soft tissues; 4, Textile material for contact with the skin; 5, Pulsed air flow textile structure; 6, (Eeach) exoskeleton's external part, made of composite material; 7, Metallic insertion; 8, Tubular seating for pull and respectively, electric cables; 9, Fasten system. Reproduced with permission from the publisher (Onose et al., 2012a, pp. 1–99).

In our perspectives from 2007 to 2008 (Figures 3, **5**, 6), we conceived a soft cable-driven exo-suit device that can apply forces to the body to assist walking. Unlike traditional exoskeletons which contain rigid framing elements, the soft exosuit is worn like clothing and uses geared motors to pull on Bowden cables connected to the suit near the ankle (Asbeck et al., 2013).

**Figure 4:**
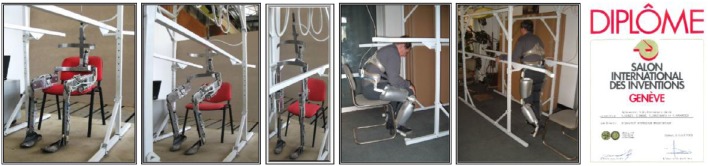
**Mechatronic Orthotic Device (MOD) Left: some relevant images of exoskeletons**. Right: Award Diploma by the Geneva International Inventions Fair. Reproduced with permission from the publisher (Onose et al., 2012a, pp. 1–99).

Some of the authors of this article have been involved in extensive scientific research on this topic for almost 15 years (Onose et al., 2008, 2012a), and were awarded with the Gold Medal at the Inventions Saloon, in Genève, Switzerland, in 2008 (Figure 4).

**Figure 5:**
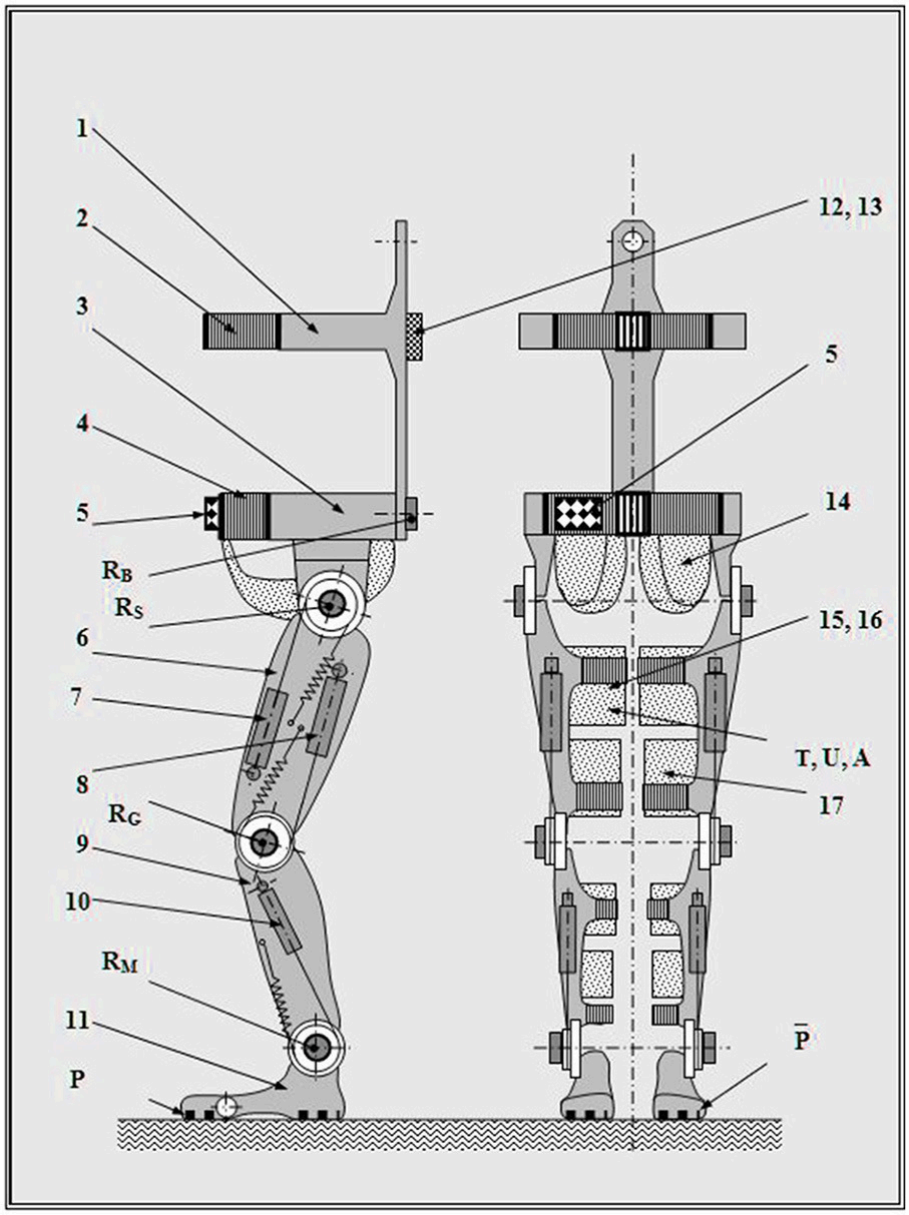
**General concept design of the MOD**. 1, Back segment; 2, Chest belt; 3, Pelvis segment; 4, Pelvis belt; 5, Programs command box; 6, Thigh segment; 7, Hip actuator; 8, Knee actuator; 9, Ankle segment; 10, Foot actuator; 11, Foot segment; 12, Connections box; 13, Pneumatic connect fitting; 14, Bottom holder; 15, Drawer panty hose; 16, Pulsed air flow textile structure; 17, Attachment belt; R, Angular transducers (B, lumbar sacral; S, hip; G, knee; M, ankle); P, Pressure sensor; T, Temperature sensor; U, Humidity sensor; A, Compressed air pressure sensor. Reproduced with permission from the publisher (Onose et al., 2012a, pp. 1–99).

We have not finished all the necessary tests and improvements of our prototype (Figure 5). Because of this delay, several mechatronic wearable powered exoskeletons are in overall more advanced stage of development, including the clinical trials.

**Figure 6:**
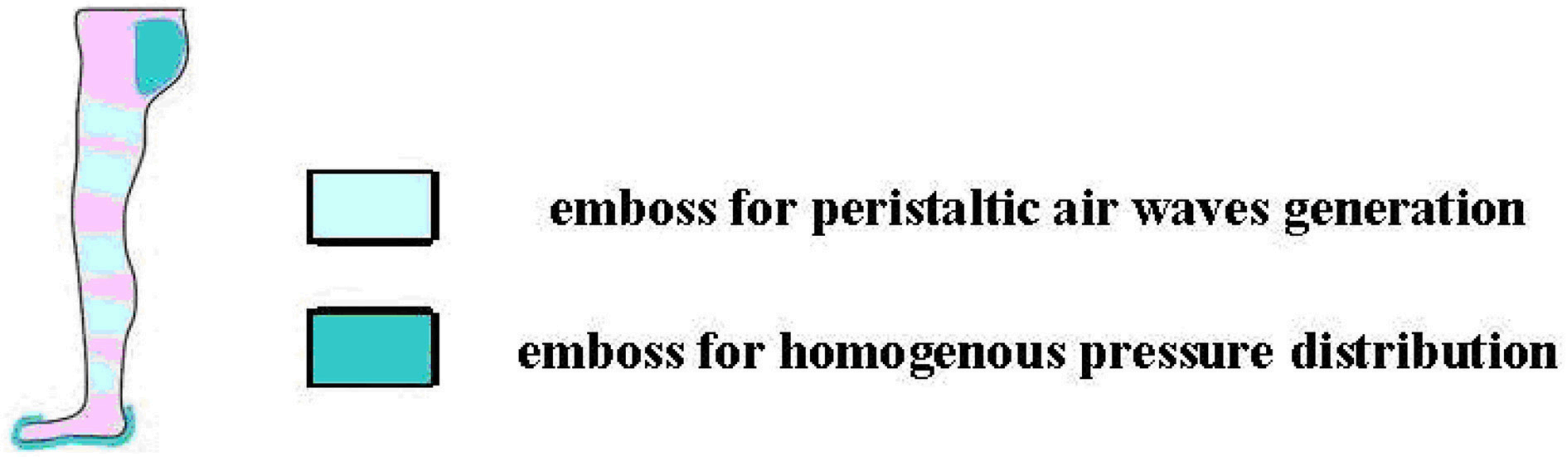
**MOD: plantar skin protection and pulsed air flow to mimic the “muscles' pump”—thus compensating the returning, venous-lymphatic circulation, in the lower limbs—concepts**. Reproduced with permission from the publisher (Onose et al., 2012a, pp. 1–99).

We re-emphasize ichnographically two important requirements to exoskeletons: (i) prevention of venous-lymphatic stasis in the lower limbs, and (ii) skin protection at the interface with the rigid structure of the exoskeleton.

Both issues can cause serious hurdles, especially during the long-time wearing of the assistive devices. Regarding the former issue, because of an impaired venous-lymphatic outflow in paralyzed persons (as well as patients with cardiovascular problems) (Guyton and Hall, 2006), regaining vertical posture with the aid of a wearable exoskeleton provokes regional stasis. This may cause discomfort and edema, including the risk of venous thrombosis, associated trophic somatic lesions, and secondary reactive heart conditions. As for the latter, an improper interface between the exoskeleton and the limb can result in mechanically generated lesions of the heel, respectively of the skin and subjacent soft tissue, which thus need for protection at the interface with the rigid structure of such devices (compressive, friction and tensile forces), as determined through performance metrics (van der Kooij et al., 2006). Therefore, we have conceived—for counteracting these problems—two components of our MOD.

Furthermore, regarding the underwear exoskeleton, we consider it ambitious and difficult, but worth trying developmental direction. Such an underwear exoskeleton would require a soft supporting structure actuated by strengthening/relaxing device. Specifically, we have foreseen, since 2006–2007 (Onose, 2007) the usage of electro-active polymers (EAPs). EAP materials seem to be the most applicable because of their large actuation forces and highest robustness (Bar-Cohen, 2005). These materials could be improved to act as artificial muscles (Bar-Cohen, 2005; Mirfakhrai et al., 2007).

The development of such a device would be a revolutionary breakthrough, since the soft support structure in cadence with the artificial muscle contraction could not only move the lower limbs but also apply thrusts to the user's calf and thighs, thus mimicking a “muscle pump” and compensating for the returning, venous-lymphatic circulation (Onose, 2007; Onose et al., 2012a). However, during the last decade not enough progress has been made in the development and subsequent translational implementation of EAPs. We still need to improve the safety, controllability and energy supply for EAPs^25^.

Another extremely challenging and still incompletely solved item, as mentioned above, is the problem of balance maintenance using advanced mechatronic wearable exoskeletons. Reliable balance is needed for prevention of falls during exoskeleton usage.

At least regarding the most difficult and demanding—in need for total assistance from the apparatus—but, at the same time, an essential category of potential beneficiaries, *the complete paraplegics*, all current devices require supplementary aids for securing the balance: walkers, crutches or—at best—canes. In sum, the powered exoskeletons that assist over-ground walking require the user to maintain balance (Swinnen et al., 2014; Yoshimoto et al., 2015; Louie and Eng, 2016).

As an illustration of the current state of this problem, it is worth mentioning a study that reported loss of balance (LOB) and falls as the primary safety outcomes (Kolakowsky-Hayner et al., 2013). Additionally, the EU FP7 “BALANCE” project (Veneman, 2014) made an observation that “none of the devices available on the market use the exoskeleton itself to support postural balance.” Therefore, the goal of the BALANCE project is to research the possibility of using the exoskeleton to maintain balance and prevent falls. The project is organized as a 4 year Specific Targeted Research Project (STREP)^26^ and has the practical objectives to “create a human-cooperative robotic postural balance controller framework,” in order to “implement the human-cooperative postural balance controller on a real exoskeleton” and to “evaluate the developed concepts in subjects walking with the exoskeleton.”^27^

Lastly, one more direction to improve the performances of a mechatronic wearable exoskeleton, is to empower it with brain's voluntary motor commands using a brain-machine-interface (BMI) (Onose et al., 2012b; Lebedev, 2014). The feasibility of such a BMI has been demonstrated in an experiment conducted in rhesus monkeys (Fitzsimmons et al., 2009). In this study, monkeys were implanted with invasive multielectrode arrays in the leg representation of the sensorimotor cortex. The animals were trained to walk bipedally on a treadmill. Activity of several hundred cortical neurons was recorded and converted into the kinematics of lower limb movements. This BMI was able to extract lower limb kinematics when monkeys walked both forward and backward on the treadmill.

These results justified the foundation of the Walk Again Project with the goal of the advancement of BMI-controlled exoskeletons (Nicolelis and Lebedev, 2009). An European project, called Mindwalker (Figure 2A), declared the goal of controlling an exoskeleton with electroencephalographic (EEG) and electromyographis (EMG) recordings (Cheron et al., 2012). Decoding of leg movements from EEGs have been demonstrated in humans walking on a treadmill (Presacco et al., 2012). Moreover, a BMI that decoded steady-state visual evoked potentials (SSVEP) from EEGs was employed to operate a lower-limb exoskeleton by healthy human subjects (Kwak et al., 2015).

The progress in BMIs for bipedal walking opens the perspective of neural control of exoskeleton-assisted walking by a completely paralyzed subject. While a slow control of walking could be provided by EEG-based BMIs, invasive BMIs hold promise to enable faster and more accurate control. However, invasive recordings are risky, so there have been no studies of invasive BMIs for walking control in humans. Additionally, it is unclear whether patients could become proficient enough to efficiently control an exoskeleton through an invasive or noinvasive BMI (Onose et al., 2012b). In this respect, we are satisfied of having deployed a clinical trial in the motor imagery BMI domain, by enrolling nine chronic tetraplegics volunteers (the largest lot for such type of trial, at that time), to control wirelessly a robotic arm by EEG commands (Onose et al., 2012b).

We have foreseen also the possibility of empowering MOD's actuating systems by voluntary commands extracted from EEGs (Onose et al., 2008). From this point of view, we have expressed our confidence in the success of the Walk Again^28^ and Mindwalker projects^29^ (Figure 2).

## Conclusion

We suggest that the joint perspective of health professionals, manufacturers and consumers on the mechatronic wearable exoskeletons should contribute to a comprehensive agenda regarding the development of devices that assist and rehabilitate bipedal posture and walking to severely impaired people. The technological advances, together with collaborative endeavors of multidisciplinary teams of researchers, could foster the progress and pave the way to the long awaited optimized and practical exoskeleton.

## Author contributions

All authors listed, have made substantial, direct and intellectual contribution to the work, and approved it for publication.

## Disclosures

GO, VC, ŞC, and VA have been the main researchers involved in achieving the aforementioned Romanian original experimental prototype of mechatronic orthotic device (MOD), but as it could be determined throughout this article, there hasn't been any intention at all to favor in description this model of device, including with the fact that it has been honestly acknowledged in the body text that currently, there are a series of other such models significantly closer to reach the optimal, desired form.

### Conflict of interest statement

The authors declare that the research was conducted in the absence of any commercial or financial relationships that could be construed as a potential conflict of interest.

## Footnotes

^1^http://www.sci-info-pages.com/other_issues.html#Osteoporosis

^2^http://www.cyberdyne.jp/company/download/20160212_financialresultssummary.pdf

^9^http://www.fastcodesign.com/3056049/a-budget-exoskeleton-allows-parapalegics-to-walk-for-the-price-of-a-car7

^10^http://www.b-temia.com/category/press_releases/

^11^https://www.cadth.ca/sites/default/files/pdf/htis/july-2015/RA0784%20Powered%20Wearable%20Walking%20Assistive%20Devices%20Final.pdf

^12^http://www.keeogo.com/biomechatronics/

^13^http://bleex.me.berkeley.edu/research/exoskeleton/bleex/

^15^http://bleex.me.berkeley.edu/research/exoskeleton/hulc/

^16^http://www.b-temia.com/defense-security-soldier-protection/

^17^http://www.cyberdyne.jp/english/products/Lumbar_LaborSupport.html

^18^http://asimo.honda.com/innovations/feature/body-weight-support-assist/

^19^http://www.scribd.com/doc/37064936/2006-Classif-Worksheet

^20^http://who.int/disabilities/policies/actionplan/disability_action_plan_en.docx

^21^http://www.keeogo.com/keeogo-in-action/

^22^http://www.cdc.gov/Features/OlderAmericans/

^23^http://www.exoatlet.com/

^24^http://www.parker.com/literature/Exoskeleton/Parker%20Indego%20Brochure.pdf

^25^http://sbdi.co.kr/cart/data/info/IDTechEx_Electroactive_Polymers_and_Devices_2013-2018_Sample.pdf?ckattempt=1

^26^http://balance-fp7.eu/project_structure.php

^27^http://balance-fp7.eu/objectives.php

^28^http://virtualreality.duke.edu/project/walk-again-project/

^29^https://www.utwente.nl/ctw/bw/research/projects/MINDWALKER/
